# Ionic liquid-assisted cellulose coating of chitosan hydrogel beads and their application as drug carriers

**DOI:** 10.1038/s41598-020-70900-7

**Published:** 2020-08-17

**Authors:** Myung-Hee Song, Thi Phuong Thuy Pham, Yeoung-Sang Yun

**Affiliations:** 1 School of Chemical Engineering, Jeonbuk National University (formerly Chonbuk National University), Baekje-daero, Jeonju-si, Jeollabuk-do 54896 Republic of Korea; 2 Faculty of Biotechnology, Ho Chi Minh City University of Food Industry, Ho Chi Minh City, Vietnam

**Keywords:** Gels and hydrogels, Ionic liquids

## Abstract

The present study proposes a simple yet effective method of cellulose coating onto chitosan (CS) hydrogel beads and application thereof as drug carriers. The beads were coated with cellulose dissolved in 1-ethyl-3-methylimidazolium acetate, an ionic liquid (IL) via a one-pot one-step process. Water molecules present in the CS beads diffused outward upon contact with the cellulose–IL mixture and acted as an anti-solvent. This allowed the surface of the beads to be coated with the regenerated cellulose. The regenerated cellulose was characterized by FE-SEM, FT-IR, and XRD analyses. To test potential application of the cellulose-coated CS hydrogel beads as a drug carrier, verapamil hydrochloride (VRP), used as a model drug, was impregnated into the beads. When the VRP-impregnated beads were immersed in the simulated gastric fluid (pH 1.2), the VRP was released in an almost ideal linear pattern. This easily fabricated cellulose-coated CS beads showed the possibility for application as carriers for drug release control.

## Introduction

Hydrogels are natural or synthetic hydrophilic structures capable of holding significant amounts of water or biological fluids in their three-dimensional structures^1–3^. Hydrogels manufactured from renewable natural resources like polysaccharides being extensively used in the medical and pharmaceutical fields^4–6^. Especially hydrogels made up of chitosan (CS), the cationic polysaccharide is of high priority owing to their impressive properties such as biodegradability, low toxicity, and biocompatibility^7–9^. However, the use of CS as drug carriers has been limited due to its weak physical properties and low acid resistance in gastric fluid when applied in oral delivery^10–12^. Therefore, in the present study, we used cellulose as a coating layer onto the surface of CS hydrogel beads to improve their stability in biomedical applications^13,14^.

Cellulose consists of β-(1 → 4)-linked glucose units and is one of the naturally abundant vital biomass components in the earth^15,16^. Cellulose is a crucial ingredient for fabricating various products such as paper, textiles, membranes, biofuel, and value-added chemical products. However, due to the strong inter- and intra-molecular hydrogen bonding, cellulose possesses high order crystalline domains^17,18^. Therefore, cellulose does not melt and is insoluble in conventional solvents such as water, dilute acids, alkali solutions, and the common organic solvents^19^, which limits the effective utilization of cellulose. Harsh conditions (high temperature and acid conditions) are usually required to disrupt the hydrogen bond network for the successful dissolution of the cellulose. These conventional methods require high energy consumption, and the use of toxic chemicals, thus, is not suitable for application in the biomedical field. As a result of thorough investigations, ionic liquids (ILs) have been considered a promising solvent that dissolves cellulose without any formation of derivatives^20–22^.

By definition, ILs are room temperature molten salts that consist of cations and anions with melting points of less than 100 °C^23,24^. In recent years, ILs have been widely used to replace conventional organic solvents in various industrial applications. In 2002, Rogers et al.^25^ reported a method to dissolve cellulose by using ILs under mild conditions without derivatization. The dissolved cellulose after that could be solidified by using anti-solvent such as water and ethanol and regenerated in various forms such as fiber^26^ film^27^ or membrane^28^, according to the manufacturing conditions.

In this study, CS beads were coated with cellulose dissolved in IL using only water molecules contained in hydrogels (Supplementary Fig. S1). The prepared cellulose coated CS beads were characterized by Field emission scanning electron microscope (FE-SEM), Fourier transform infrared spectrometer (FT-IR), and X-ray diffractometer (XRD), and applied to drug release experiments and discussed accordingly.

## Results and discussion

### Morphology of cellulose-coated CS bead

**Figure 1:**
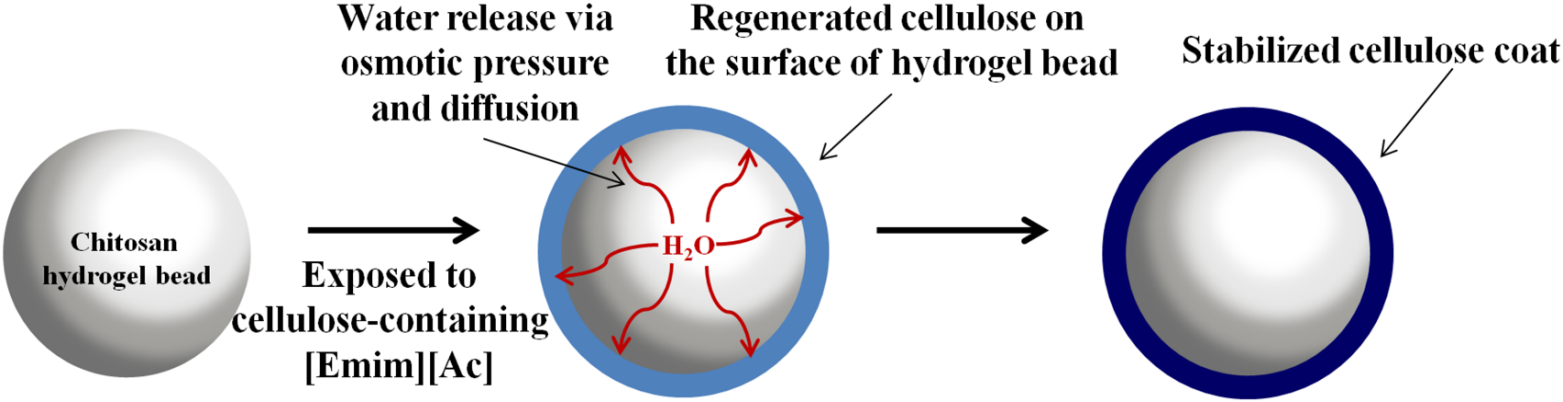
Schematic presentation of cellulose coating principle.

The conceptual strategy for the cellulose coating on hydrogel is shown in Fig. 1. The strategy was to evenly coat wet CS hydrogel bead with dissolved cellulose via osmotic pressure difference. That is, cellulose was first dissolved in [Emim][Ac], which is a well-known cellulose processing IL^29^. Then the wet CS bead, which was used as a model hydrogel, was brought into contact with the dissolved cellulose. Upon contact with the cellulose solution, the water molecules present inside of the CS bead diffused outwards due to osmotic pressure. Consequently, the diffused water molecules acted as anti-solvents to solidify the cellulose and formed cellulose coating on the surface of the hydrogel. It must be noted that control of the water content of the hydrogel bead was essential at this stage. It was observed that when the bead was too watery (having water on the outside), the cellulose coating formed was uneven, rough and tend to clump up. Moreover, when the bead was dried or had no water inside, it was impossible to form the cellulose coating due to the lack of water molecules to act as anti-solvents. Thus, the best condition for this idea to be realized was to control the moisture content of the bead by effectively wiping off surface water. For this purpose, the wet bead was placed on gauze to remove adhering water from the surface of the bead before use.

### FE-SEM analysis

**Figure 2:**
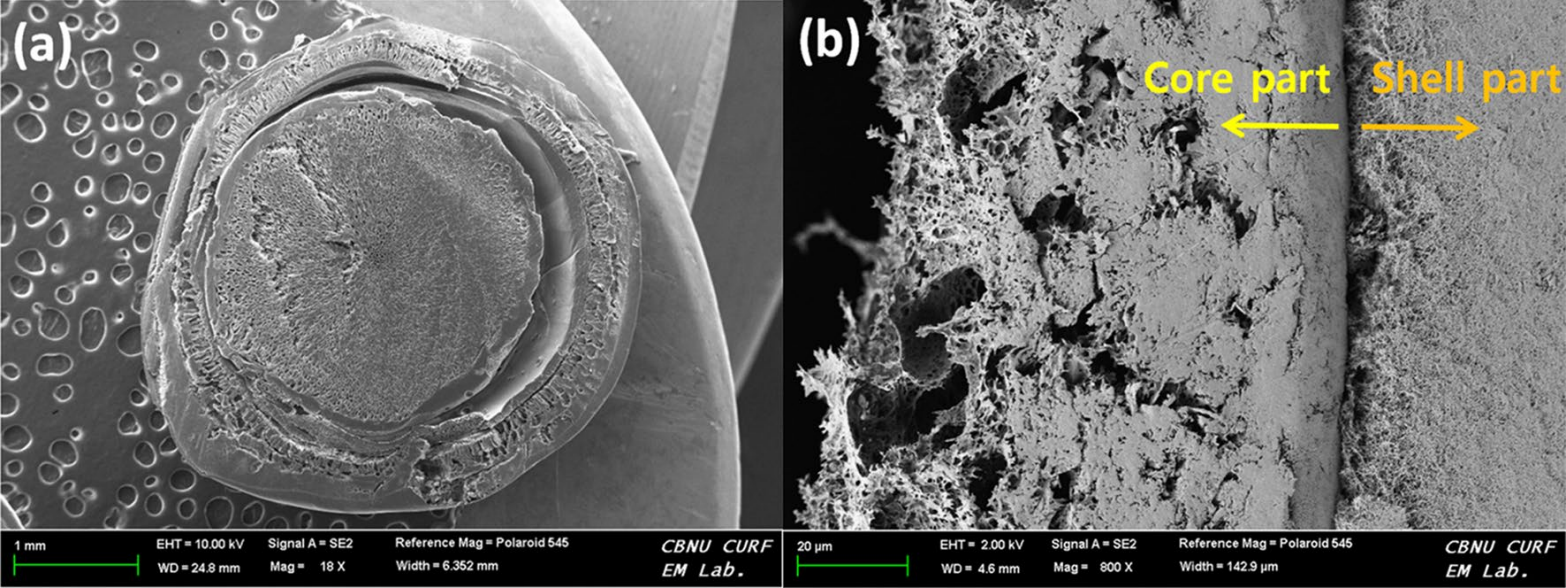
FE-SEM images of freeze dried cellulose-coated CS bead (**a**) and intersection of CS and cellulose layers (**b**). Scale bars represent 1 mm and 20 µm respectively.

**Figure 3:**
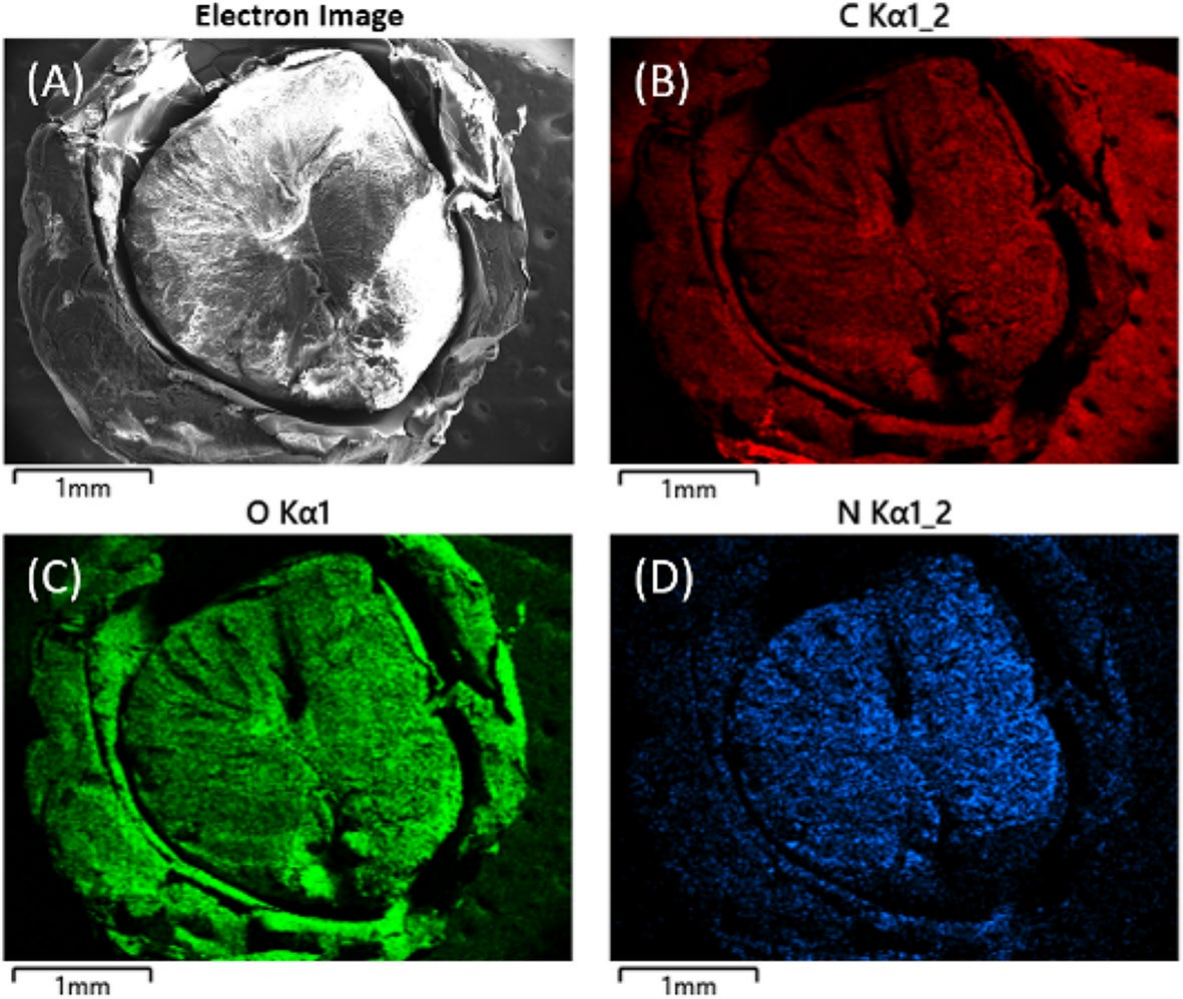
FE-SEM images of cellulose-coated CS bead (**A**) and EDS mapping of C (**B**), O (**C**), N (**D**) elements.

To understand the texture, the cellulose-coated CS bead was cut in half, and the morphology was analyzed using FE-SEM, as shown in Fig. 2. The FE-SEM images showed the apparent boundary of CS and cellulose layers (Fig. 2a). The cross-sectional internal morphology (Fig. 2b) clearly showed that regenerated cellulose appeared as an outer layer (shell), and CS hydrogel was present as the core. Higher magnification of core and shell parts showed a connected three-dimensional network (Supplementary Fig. S2). Both the core and shell parts possessed high porosities, with the shell part showing a broad range of pore sizes and the core part revealing smaller pore sizes. This porous network can be useful to encapsulate drugs or cells for biological applications. In addition, the results of FE-SEM analysis (Fig. 3A) and element mapping (Fig. 3B–D) of the cross-section of the cellulose-coated CS bead are shown in Fig. 3. Since carbon and oxygen are constituent elements of chitosan (CS) and cellulose, the elements C (Fig. 3B) and O (Fig. 3C) were evenly distributed throughout the cellulose-coated CS bead. However, it was confirmed that the elements of N (Fig. 3D) are concentrated in the core (chitosan part). Additionally, as a result of EDS mapping by separating the core part and the shell part, C, O, N, and Na were measured as 70.32, 25.71, 3.88, 0.09 wt% and 53.64, 46.28, 0.00, 0.08 wt%, respectively in the core and shell parts (Table 1). That is, the N element was detected only in the core, the chitosan portion. Therefore, it was confirmed that the core is composed of chitosan and the shell is composed of cellulose. In addition, if some of the dissociated [Emim][Ac] remains in cellulose-coated CS bead, N, a constituent element of [Emim][Ac], must be detected not only in the core but also in the shell part. However, since the cellulose-coated CS beads were thoroughly washed, no ionic liquid residues were considered.

**Table 1:** The contents (wt%) of C, O, N, Na elements of chitosan and cellulose part of cellulose-coated CS bead analyzed by FE-SEM EDS mapping.

Element	Chitosan part (wt%)	Cellulose part (wt%)
C	70.32	53.64
O	25.71	46.28
**N**	3.88	0.00
Na	0.09	0.08
Total	100.00	100.00

### FT-IR analysis

**Figure 4:**
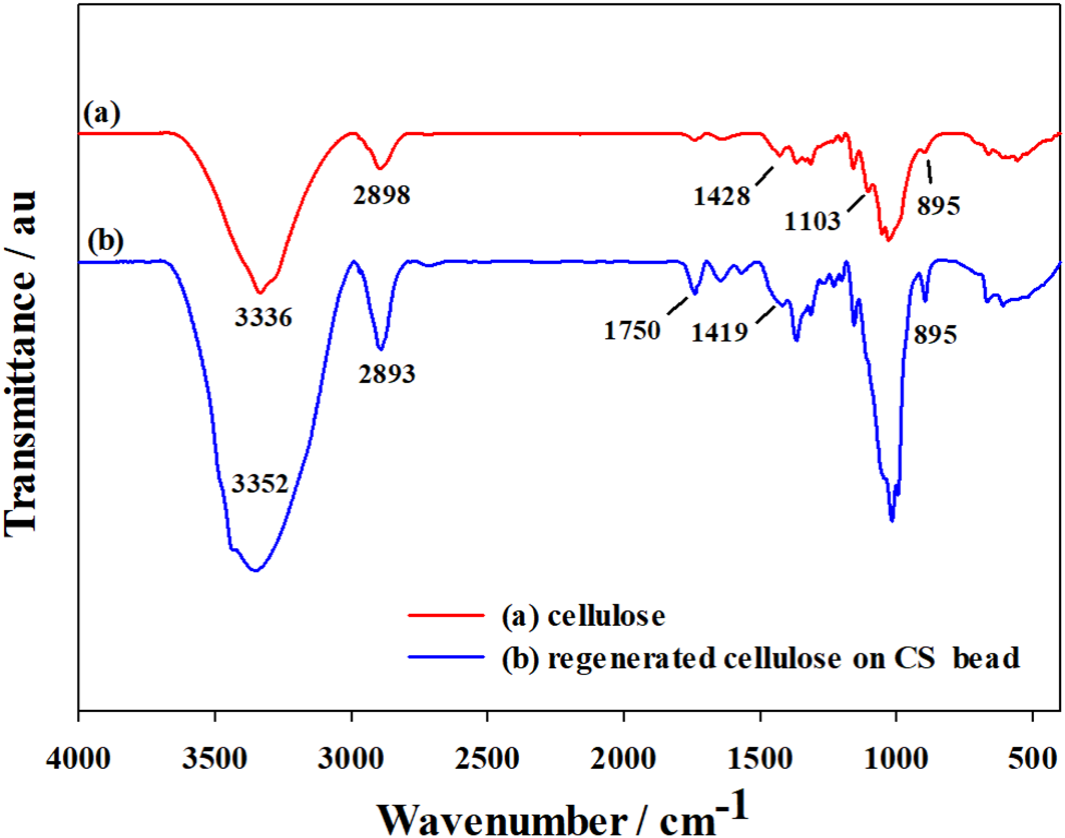
FT-IR spectral data of original cellulose (**a**) and regenerated cellulose on CS bead (**b**). Original cellulose was in powder form and regenerated cellulose was separated from cellulose-coated CS bead.

Prior to FT-IR analysis, the cellulose-coated CS bead was separated into the shell (regenerated cellulose) and core (CS) part to confirm the functional groups of each section. FT-IR spectroscopic analysis of the original cellulose and regenerated cellulose on CS bead are shown in Fig. 4. The broad absorption band in the range of 3,300–3,400 cm^−1^ was due to the intermolecular O–H stretching vibration of the cellulose molecule. The peak at 2,898 cm^−1^ observed for native cellulose was due to the C–H stretching vibration of CH_2_ and CH_3_ groups. This band was not affected by changes of crystallinity; thus, there was no significant change of this band in the regenerated cellulose spectral data. The peak at 1,428 cm^−1^ in the original cellulose was assigned to the crystallized cellulose I and amorphous cellulose, whereas in the case of the regenerated cellulose, this band shifted to 1,419 cm^−1^, representing cellulose II and amorphous cellulose^29,30^. The peak at 1,428 cm^−1^ in the cellulose spectra was assigned to symmetric CH_2_ bending vibration. The peak at 1,103 cm^−1^, which appeared in the spectrum of the native cellulose was not observed for the regenerated cellulose. This further confirmed the prevalence of crystalline cellulose II^7^. In the regenerated cellulose spectra, the new peak at 1,750 cm^−1^ was attributed to the C=O in the acetate^31^. This is good evidence that cellulose dissolved in [Emim] [Ac] had been regenerated. The band observed at around 895 cm^−1^ was due to the stretching vibration of C–O bond in the amorphous regions of cellulose and regenerated cellulose.

### XRD analysis

**Figure 5:**
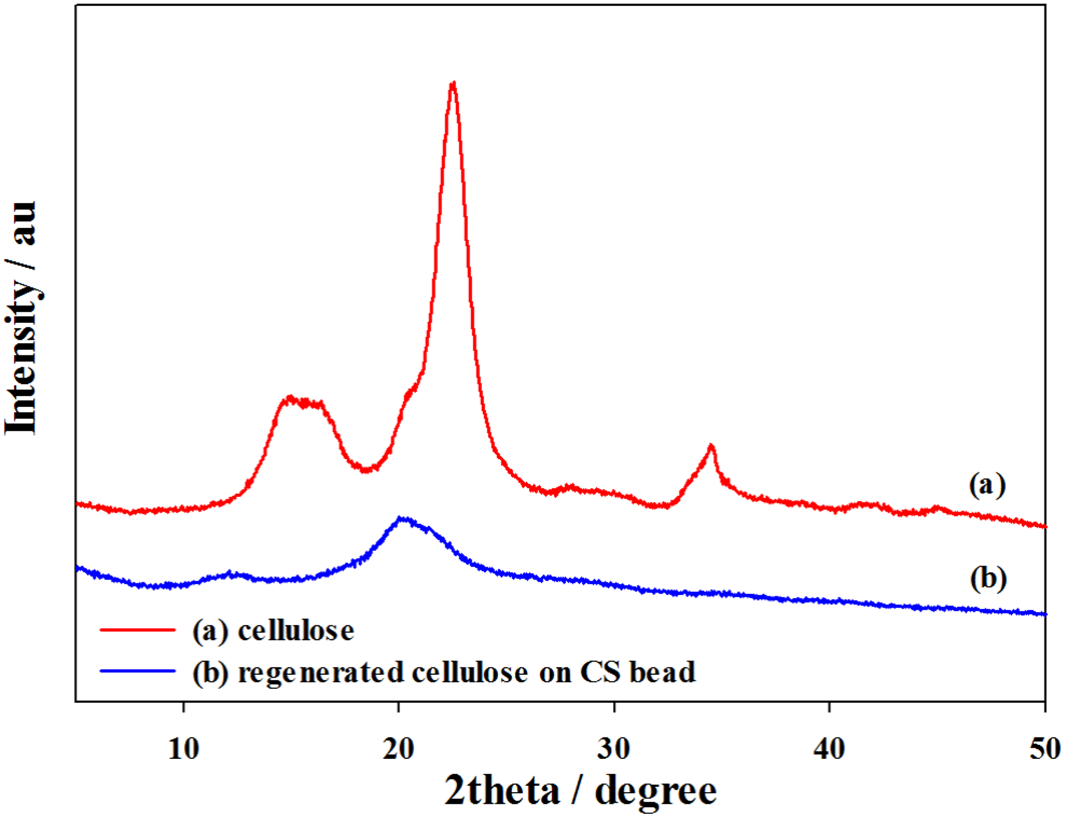
XRD profiles of original cellulose (**a**) and regenerated cellulose on CS bead (**b**). Original cellulose was in powder form and regenerated cellulose was separated from cellulose-coated CS bead.

The typical XRD profiles of the original cellulose and regenerated cellulose on CS bead are shown in Fig. 5. The XRD pattern of original cellulose exhibits characteristic diffraction peaks at 2θ = 14.9°, 16.0°, 22.4°, and 34.5° corresponding to the planes (110), (110), (200) and (400). These diffraction patterns confirmed that the original cellulose was cellulose I. After dissolution and regeneration in [Emim][Ac] the regenerated cellulose exhibited characteristic diffraction patterns at 2θ 19°–20°, and these patterns were consistent with cellulose II. Similar results were reported with other IL solvents such as [Bmim]Cl, [Amim]Cl^32,33^. Due to the pronounced quantity of amorphous cellulose existing in the regenerated cellulose, it showed a lower crystallinity than the original cellulose. It could be explained by the fact that [Emim][Ac] broke the inter- and intramolecular hydrogen bonds and reduced the crystallinity of the original cellulose during the dissolution process.

### In vitro release of VPR from CS beads and cellulose-coated CS beads

Since cellulose coatings may improve the physical and chemical stability of the hydrogels, cellulose-coated CS beads may have a wide range of applications. One of the attractive applications of the cellulose-coated hydrogels could be in the biomedical area because both cellulose and CS are biocompatible and biodegradable.

**Figure 6:**
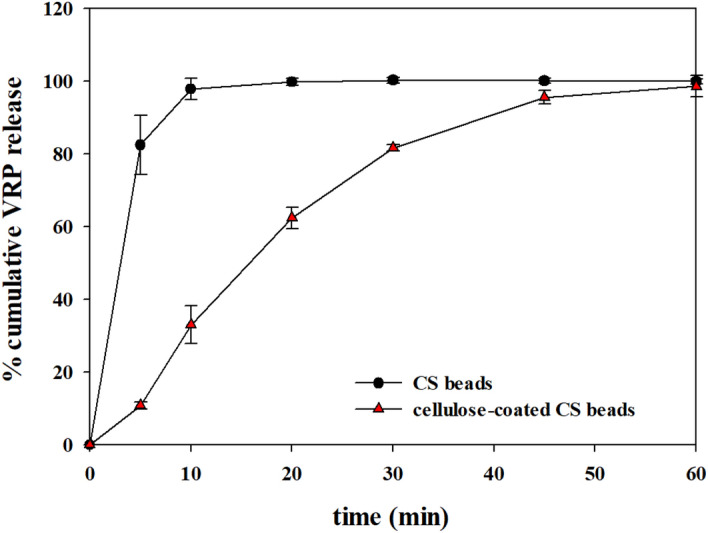
Release patterns of VRP in SGF from CS beads and cellulose-coated CS beads. The in vitro release experiment was performed at 100 rpm and 37 ± 0.5 °C. The concentration of VRP was analyzed by taking a sample of 0.5 ml from 50 ml of SGF solution.

**Figure 7:**
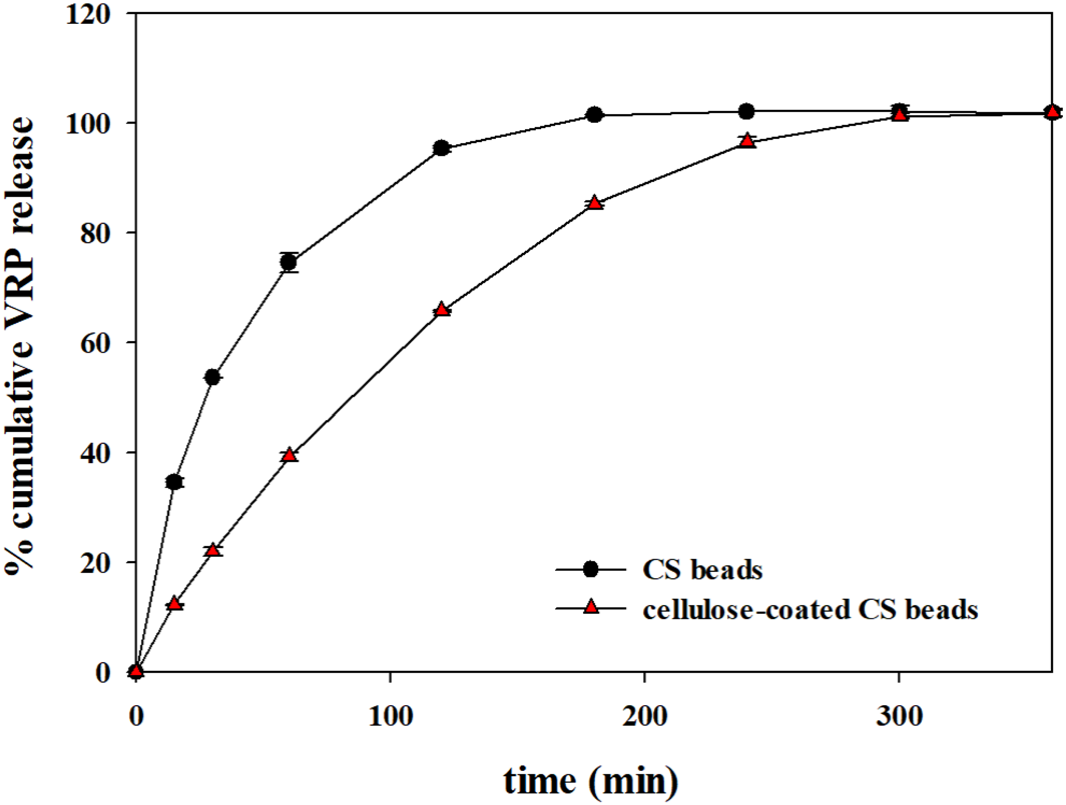
Release patterns of VRP in SIF from CS beads and cellulose-coated CS beads. The in vitro release experiment was performed at 100 rpm and 37 ± 0.5 °C. The concentration of VRP was analyzed by taking a sample of 0.5 ml from 50 ml of SIF solution.

Hence we tested the cellulose-coated CS beads for in vitro verapamil hydrochloride (VRP) release applications. VRP is a calcium channel blocker used for the treatment of hypertension, angina and myocardial infarction^34^. The release experiments were studied in different release fluids, including simulated gastric fluid (SGF, pH 1.2) and simulated intestinal fluid (SIF, pH 6.8). The release profiles corresponding to a formulation consisting of CS beads and cellulose-coated CS beads are displayed in Figs. 6 and 7 for SGF and SIF, respectively. As shown in Fig. 6, the release patterns of VRP from the cellulose-coated CS beads in SGF were significantly different from those of CS beads. This could be explained by the stability of the prepared CS hydrogels. In SGF, CS beads were dissolved only within 5 min, and no solid matter was left after 10 min in the system. Meanwhile, the cellulose-coated CS beads were stable and remained stable up to 24 h. The release of VRP from the cellulose-coated CS beads was almost linear, similar to the zero-order release.

Figure 7 shows the VRP release profiles of the CS beads and cellulose-coated CS beads in SIF. The release of VRP from the CS beads was much faster than that from the cellulose-coated CS beads. After 120 min, 95 ± 0.5% and 65 ± 0.2% of VRP were released from the CS beads and cellulose-coated CS beads, respectively, indicating that the cellulose coating makes a significant change in VRP release profiles. The total amounts of drug released from both coated and uncoated CS beads after 300 min were nearly the same. The results of this study were tried to be applied to the well-explained drug release model Korsemeyer–Peppas model (supplementary information). The release exponent (*n*) for CS beads are smaller than 0.45 in SGF (0.0752) and SIF (0.3217), indicating that the release of VRP from the CS beads are a Quasi-Fickian diffusion mechanism (Supplementary Table S1). This mechanism indicated that VRP diffuses partially through a swollen matrix or its pores in the chitosan hydrogels. Moreover, the *n* values of cellulose-coated CS beads in SGF and SIF were 0.7298 and 0.6628, respectively, which indicated the non-Fickian diffusion mechanism consisting of a combination of diffusion and polymer relaxation. By coating cellulose on CS beads, it was possible to alter the release pattern of the drugs in the CS beads. These results clearly demonstrated the potential application of the prepared cellulose-coated CS hydrogel beads in the biomedical area of drug delivery.

## Conclusions

Cellulose-coated CS beads were successfully fabricated via a facile one-pot one-step method. FE-SEM observation showed that the CS beads and the cellulose coating layer were separated and porous. The results of FT-IR and XRD also supported that the regenerated cellulose was well coated on the CS surface. The cellulose-coated CS beads exhibited sustained release patterns of VRP in SGF and SIF environments when applied as drug carriers. This simple cellulose coating method will be able to promote various applications of the hydrogels.

## Experimental

### Chemicals and materials

Cellulose (powder) and verapamil hydrochloride (VRP) were purchased from Fluka and Sigma-Aldrich, respectively. CS (viscosity at 20 °C: 200–220 cP, deacetylation degree: 85.9%) was purchased from YBBio Co., Ltd. (Korea), at which CS was obtained on an industrial scale by the deacetylation of chitin from the red crab. The ILs 1-Ethyl-3-methylimidazolium acetate ([Emim][Ac], above 95%) was purchased from Ionic Liquids Technologies (Germany), and acetic acid (above 99.7%) was obtained from Junsei Chemical Co., Ltd. All of the other reagents used in this study were also of analytical grade. In addition, the plastic hub needle used to fabricate the CS beads were purchased from Teaha Co., Korea.

### Dissolution of cellulose in ionic liquid

Cellulose/[Emim][Ac] solution was first prepared by dissolving 6 g of cellulose powder in 100 ml of [Emim][Ac] at 120 °C, and the obtained 6% (w/v) cellulose/[Emim][Ac] solution was used to manufacture the regenerated cellulose-coated CS beads.

### Fabrication of CS beads

For the manufacture of the CS beads, CS powder (2 g) was dispersed in 100 ml of distilled water containing 2 ml of acetic acid solution at room temperature. The mixture was left overnight with continuous mechanical stirring to prepare completely dissolved CS solution. The as-obtained mixture was dropped through a plastic hub needle with a diameter of 310 µm into 1 M NaOH solution under gentle magnetic stirring at room temperature to obtain CS beads. After 2 h, the hydrogel beads were separated from the NaOH solution and washed with distilled water.

### Fabrication of VRP-loaded CS beads

To fabricate the VRP-loaded CS beads, 1,000 mg of VRP was added to 10 ml of 2% CS solution and stirred for 30 min to obtain a homogeneous dispersion. The VRP-loaded CS beads were prepared in the same dropwise method, as described in the previous section.

### Procedure for cellulose coating on CS beads

The wet CS beads were dropped into the 6% (w/v) cellulose dissolved in [Emim][Ac] solution and kept for 5 min, resulting in the formation of cellulose coat on the surface of CS beads. Then, the cellulose-coated CS beads were separated from the solution using gauze mesh and washed with deionized water three or more times.

### Fabrication of VRP-loaded cellulose-coated CS beads

The VRP-loaded cellulose-coated CS beads were fabricated by first manufacturing the VRP-loaded CS beads and then coating it with cellulose. The verapamil encapsulation efficiency (EE) and loading capacity (LC) of cellulose-coated CS beads used in drug release experiments were 100 ± 0.0% and 8.62 ± 0.07%, respectively. Encapsulation efficiency (EE) and loading capacity (LC) were calculated as follows:

$$ \begin{aligned} {\text{EE }}\left( {{\% }} \right) & = \frac{weight\,of\,loaded\,drug}{{weight\,of\,total\,added\,drug}} \times 100 \\ {\text{LC }}\left( {{\% }} \right) & = \frac{weight\,of\, loaded\,drug}{{weight\,of\,drug\,loaded\,bead}} \times 100. \\ \end{aligned} $$

### Drug release studies

The in vitro release studies were performed in simulated gastric fluid (SGF, pH 1.2) and simulated intestinal fluid (SIF, pH 6.8). Ten beads were placed in plastic bottles containing 50 ml of the release medium. After the cellulose coating, the weight per bead was increased by cellulose coating, so the number of beads was used instead of the weight of beads. The drug release experiments were carried out at 100 rpm and 37 ± 0.5 °C in a shaker. At predetermined time intervals, samples of 0.5 ml were collected from the release medium and replaced with fresh SGF and SIF solution, respectively. The experiments were duplicated.

To estimate the initial concentration of VRP entrapped into the beads, the VRP concentration was measured after crushing and dissolving 10 VRP-loaded CS beads in 50 ml of medium, which is the same condition as the release experiment.

$$ \% \,{\text{cumulative}}\,{\text{VRP}}\,{\text{release}} = { }\frac{{{\text{conc}}.{ }\,{\text{of}}\,{\text{VRP}}}}{{{\text{initial}}\,{\text{conc}}.\,{\text{of}}\,{\text{VRP}}}}{ } \times { }100. $$

### Instrumental details

In the dried cellulose-coated CS bead, the core and the shell part are easily separated. In this study, a cross-section was cut using a stationery knife and tweezers and then FE-SEM analysis was performed, and FT-IR and XRD analyses were performed by separating the core and shell. The morphology of the core (CS) and shell (cellulose) parts of the cellulose-coated CS beads was examined using an FE-SEM (Carl Zeiss, SUPRA 40VP, Germany) equipment. FT-IR (Spectrum GX, Perkin Elmer, USA) was used to obtain the functional groups present in the fabricated materials. Multipurpose high-performance X-ray diffractometer (XRD, X'pert Powder, PANalytical, Netherlands) was used to analyze the crystallographic nature of the samples. Furthermore, the concentration of drug in the solution was assayed by using high-performance liquid chromatography (HPLC, Shimadzu CBM-20A, Kyoto, Japan) at 278 nm. The mobile phase condition was 75% methanol and 25% buffer solution of 25 mM ammonium formate. The used column was Zorbax Eclipse XDB-C18 (Agilent, USA).

## Supplementary information

Supplementary Information.
