# Supplementary material for: Ionic liquid-assisted cellulose coating of chitosan hydrogel beads and their application as drug carriers
Source: Sci Rep. 2020 Aug 17;10:13905. doi: 10.1038/s41598-020-70900-7 (PMC7431572; doi:10.1038/s41598-020-70900-7)
Supplement: Supplementary file 1 — Supplementary Information. [file 41598_2020_70900_MOESM1_ESM.docx]

Supplementary information of

Ionic liquid-assisted cellulose coating of chitosan hydrogel beads and their application as drug carriers

Myung-Hee Song^†^, Thi Phuong Thuy Pham^‡^, Yeoung-Sang Yun^†,*^

*^†^ School of Chemical Engineering, Jeonbuk National University (formerly Chonbuk National University), Baekje-daero, Jeonju-si, Jeollabuk-do, 54896, Republic of Korea*

*^‡^ Faculty of Biotechnology, Ho Chi Minh City University of Food Industry, Ho Chi Minh City, Vietnam*

| 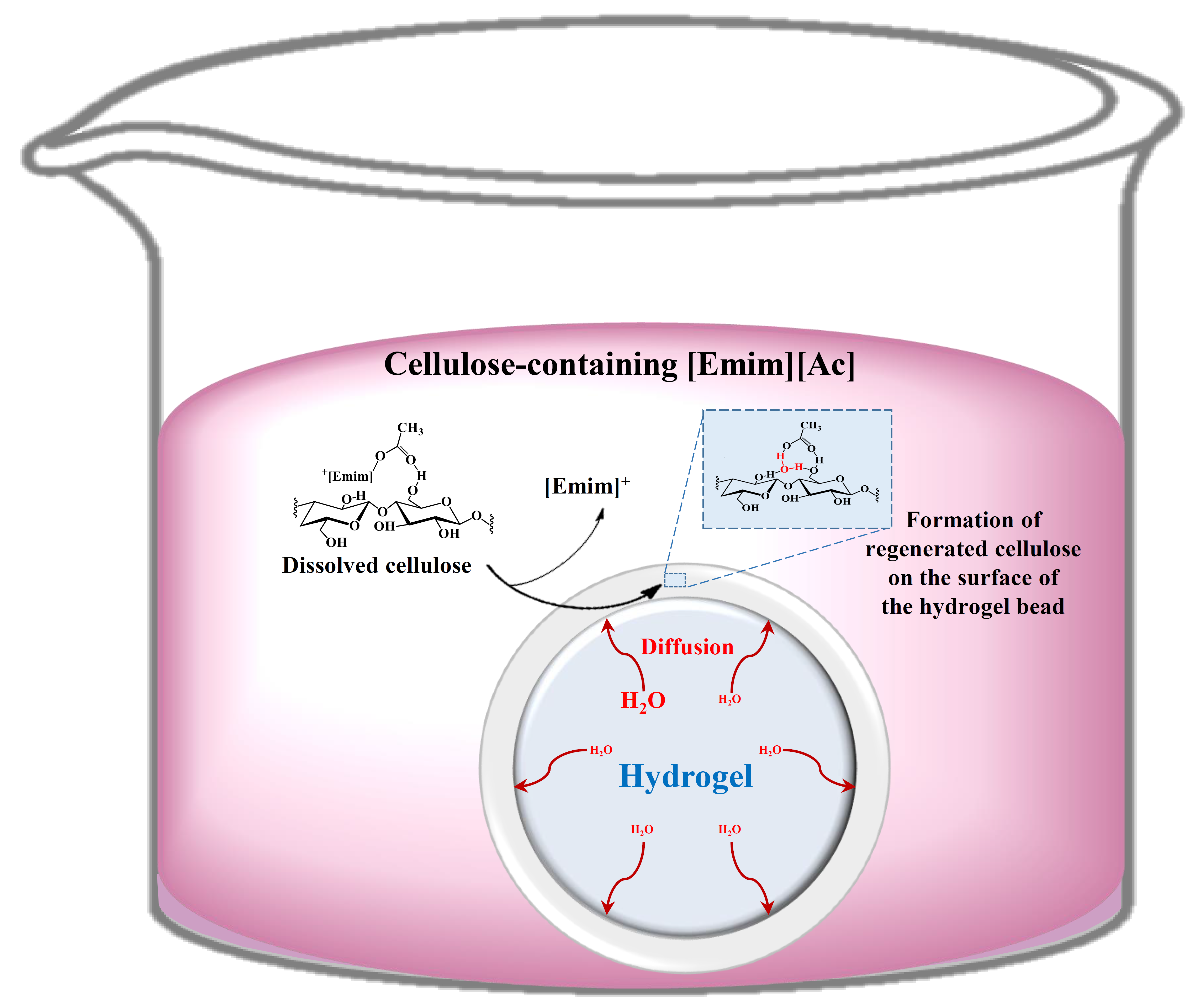 |
| --- |
| **Fig. S1** Schematic diagram of cellulose coating by water molecules contained in the hydrogel |

| 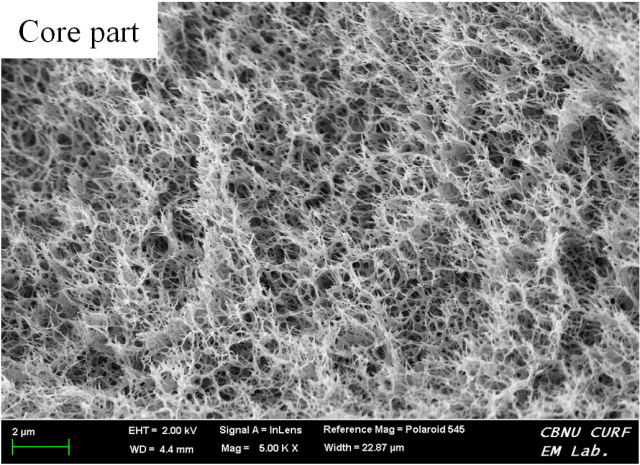 | 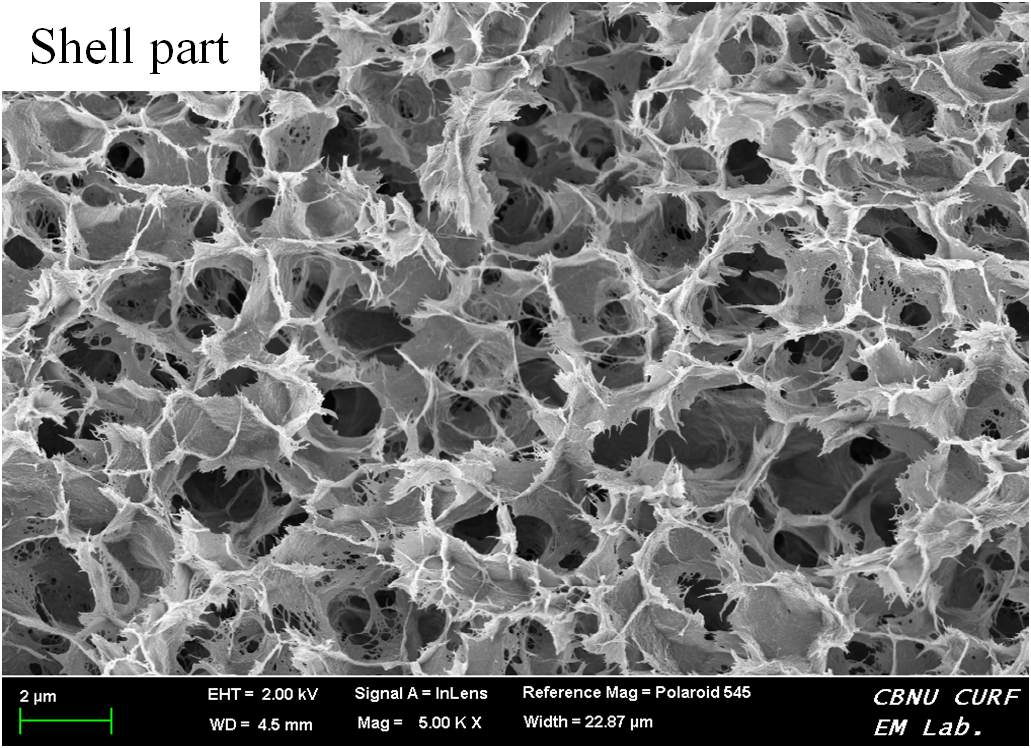 |
| --- | --- |
| **Fig. S2** FE-SEM images of core (CS) part and shell (cellulose) part of freeze dried cellulose-coated CS bead | |

Table S1. *In vitro* diffusion exponent parameters from Korsemeyer-Peppas model of VPR-loaded CS bead and cellulose-coated CS bead

| Condition | Type | Diffusion exponent (*n*) | R^2^ | Overall solute diffusion  mechanism |
| --- | --- | --- | --- | --- |
| SGF | CS beads | 0.0752 | 0.9907 | Quasi-Fickian |
|  | Cellulose-coated CS beads | 0.7298 | 0.9727 | Anomalous diffusion or  non-Fickian diffusion |
| SIF | CS beads | 0.3217 | 0.9682 | Quasi-Fickian |
|  | Cellulose-coated CS beads | 0.6628 | 0.9929 | Anomalous diffusion or  non-Fickian diffusion |

Korsemeyer-Peppas model (Eq.1).

$\frac{M_{t}}{M_{\infty}}$ 〓 $Kt^{n}$ (Eq. 1)

*M_t_*: amount of drug released at time ‘t’

*M_∞_*: total amount of drug in dosage form

*K*: kinetic constant

*n*: diffusion or release exponent

*t*: time

For Quasi-Fickian diffusion the value of *n* < 0.45, Fickian diffusion *n* = 0.45, non-Fickain or anomalous transport *n* = 0.45 – 0.89 and Case II transport *n* > 0.89 ^1^.

**Reference**

1 Singhvi, G. & Singh, M. In-vitro drug release characterization models. *Int J Pharm Stud Res* **2**, 77-84 (2011).
